# Xanthogranulomatous Orchitis: A Case Report of a Rare Entity Affecting the Male Gonad

**DOI:** 10.7759/cureus.93924

**Published:** 2025-10-06

**Authors:** Kumar Pankaj, Rishin Dutta, Suvit Jumde, Divyanshu Joshi

**Affiliations:** 1 Urology, Swami Rama Himalayan University, Dehradun, IND

**Keywords:** e. coli, epididymo- orchitis, testicular neoplasm, touton giant cells, xanthogranulomatous orchitis

## Abstract

Xanthogranulomatous orchitis (XGO) is an uncommon chronic inflammatory condition that is characterized by testicular tissue destruction and macrophage infiltration, with a limited number of documented cases in the literature. We present a case study of a 52-year-old gentleman who initially presented with symptoms consistent with epididymo-orchitis, but later required orchiectomy due to non-viability of the right testis on Doppler ultrasound. Subsequent histopathological examination led to a diagnosis of XGO. The etiology of XGO remains unclear; however, it is speculated that infections, particularly *E. coli*, and epididymal obstruction contribute to its pathogenesis. The differentiation of XGO from testicular tumors poses a clinical challenge, as pre-operative diagnostic methods are inadequate, necessitating surgical excision for definitive diagnosis and treatment. This report emphasizes the rarity of XGO, its diagnostic complexities, and the significance of recognizing this condition in the context of differential diagnoses in testicular pathology.

## Introduction

Xanthogranulomatous orchitis (XGO) is a benign chronic inflammatory condition that has the characteristic feature of destruction of normal testicular tissue and macrophage infiltration [1]. In the genitourinary system, xanthogranulomatous inflammation most commonly occurs in the kidney, bladder, and prostate, with rare occurrence in the testis [2]. Testicular cancer is the most common differential diagnosis, with malakoplakia and Rosai-Dorfman disease being some of the other closest differentials [3]. XGO is primarily diagnosed histologically after orchiectomy. We report a case of XGO that presented with features of epididymo-orchitis.

## Case presentation

**Figure 1:**
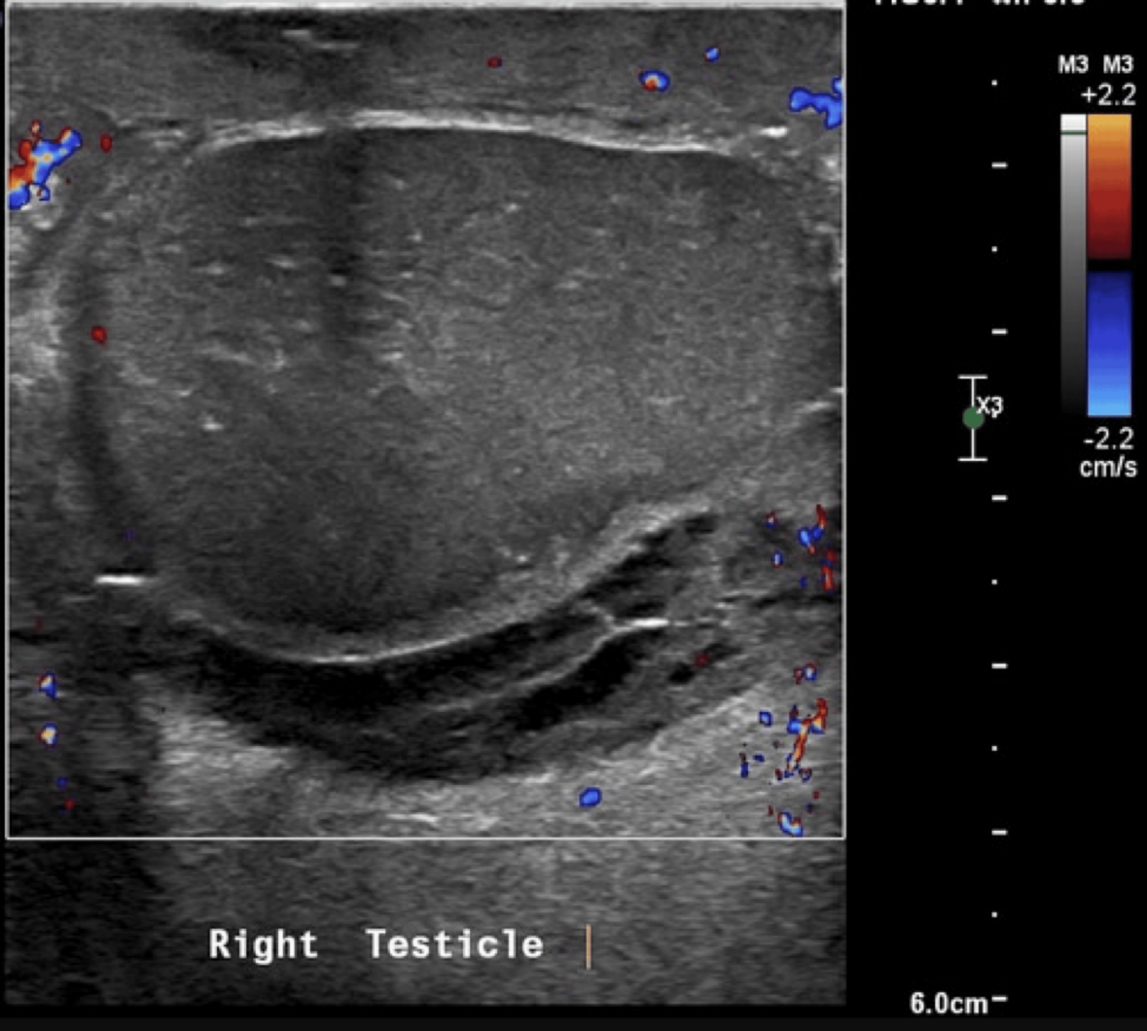
Color Doppler ultrasound of right testicle showing no vascularity

A 52-year-old gentleman presented with complaints of pain and swelling in his right testicle associated with intermittent fever and lower urinary tract symptoms. There was no previous history of trauma. On clinical examination and supportive investigations with ultrasound, urine examination, a diagnosis of right epididymo-orchitis was made, and treatment was initiated with antibiotics, anti-inflammatories, and analgesics. The patient was non-compliant to treatment and presented one month later with severe pain in the right testis and loss of vascularity on colour Doppler ultrasound (Figure 1). In view of the non-viable right testis, the patient was taken up for right orchiectomy. 

**Figure 2:**
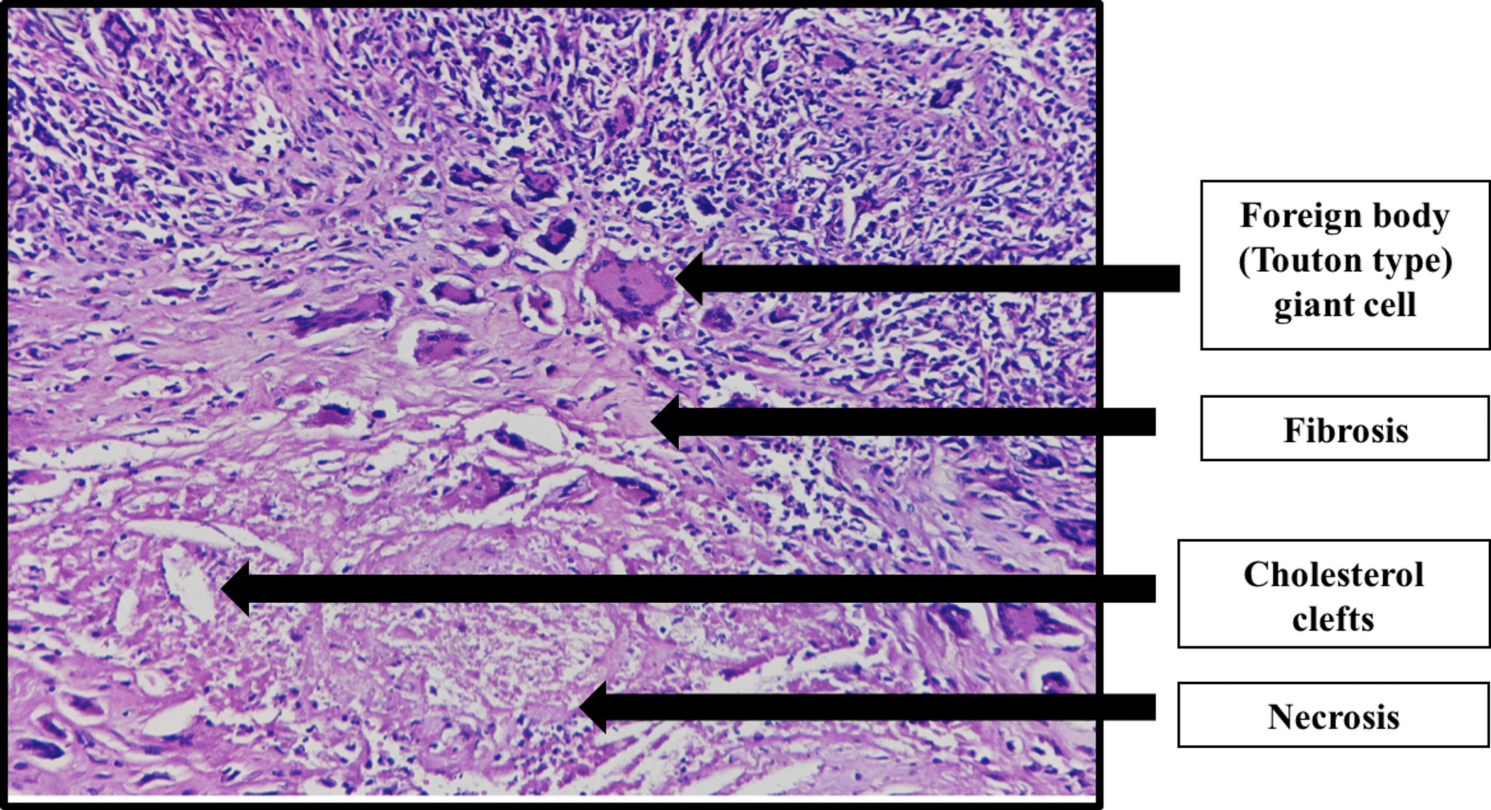
Histopathological image (in 40X magnification)

Macroscopically, the resected specimen was grey-yellow, firm, with an area measuring approximately 48 x 43 x 33 mm. On histological examination, there was the presence of lymphohistiocytic infiltrates and foamy macrophages along with numerous foreign bodies and a few Touton-type giant cells (Figure 2). Fibro-histiocytic proliferation, along with moderate mixed inflammatory infiltrates consisting predominantly of lymphocytes and plasma cells, was seen. Based on the above microscopic findings, a diagnosis of xanthogranulomatous orchitis was established.

## Discussion

The kidney is the most common genitourinary organ affected by xanthogranulomatous inflammation, whereas XGO is a rare manifestation [4]. Furthermore, most cases of XGO present with suspicion of testicular neoplasm [3]. In our case, it presented as a non-viable testis with a history of epididymo-orchitis.

Destruction of the normal testicular tissue, which is replaced by lipid-laden macrophages, is the characteristic histopathological feature of XGO [5]. The exact pathology of XGO is still unknown, and infection coupled with epididymal obstruction seems to play a major role. In all reported cases where urine culture was obtained, *E. coli *was the most common organism identified [6]. This also points to the fact that the majority of the infections were localized and not from other sources through hematogenous spread. Obstruction, failure of antegrade sperm flow, and reflux of infected urine lead to activation of the immune response, which leads to the xanthogranulomatous inflammation [6].

Spermatic tract dysfunction caused by diabetic neuropathy has also been established to play a major role in the inflammatory process [7]. In post-prostatectomy patients, Nistal et al. [7] reported xanthogranulomatous orchioepididymitis due to mechanical obstruction of the spermatic tract.

Though obstruction seems to play a major role in the pathogenesis of XGO, it is not mandatory. This is supported by a case report describing XGO following BCG instillation after TURBT in a patient with a bladder tumor [8].

The major differential diagnosis of XGO includes bacterial infection, mainly caused by anaerobic bacteria, malakoplakia, tumors of the testis, and Rosai-Dorfman disease [3]. Case reports in the literature have shown seminoma and XGO to occur in the same testis [9].

Clinically, it is often very difficult to differentiate XGO from testicular tumors, and no definitive pre-operative diagnostic methods exist. Post-operative tissue diagnosis is the only accurate diagnostic tool. Surgical excision, either complete or partial, is the definitive therapy for XGO because of its destructive nature [9]. Coupled with surgical excision, antibiotic therapy, including anaerobic coverage, is also paramount.

To date, only 23 cases of xanthogranulomatous orchitis have been reported in the literature, which have been summarised in Table 1.

**Table 1:** Summary of xanthogranulomatous orchitis cases reported in literature

Study	Title	Journal name	Year of publication
Wiener et al. [10]	Xanthogranulomatous epididymitis: a case report	Journal of Urology	1987
Usamentiaga et al. [11]	Xanthogranulomatous orchitis	Urology	1998
Vaidyanathan et al. [12]	Xanthogranulomatous funiculitis and epididymo-orchitis in a tetraplegic patient	Spinal Cord	2000
Hajri et al. [13]	Xanthogranulomatous orchitis. Report of 7 cases	Annales d’urologie	2001
Nistal et al. [7]	Xanthogranulomatous funiculitis and orchiepididymitis: report of 2 cases with immunohistochemical study and literature review	Archives of Pathology & Laboratory Medicine	2004
Yap et al. [14]	Xanthogranulomatous orchitis.	Urology	2004
Demirci et al. [15]	Xanthogranulomatous orchitis with scrotal fistulas	International Journal of Urology	2004
Salako et al. [16]	Xanthogranulomatous orchitis in an adult Nigerian	International Journal of Urology	2006
Al‐Said et al. [17]	Xanthogranulomatous orchitis: review of the published work and report of one case	International Journal of Urology	2007
Rifat et al. [18]	An unusual case of extensive xanthogranulomatous orchitis in a diabetic patient	Medical Principles and Practice	2009
Val‐Bernal et al. [19]	Concurrent xanthogranulomatous orchiepididymitis and seminoma in the same testis	Pathology international	2010
Repetto et al. [20]	Bilateral xanthogranulomatous funiculitis and orchiepididymitis in a 13-year-old adolescent boy	Journal of Pediatric Surgery	2012
Ezer et al. [21]	Xanthogranulomatous orchiepididymitis: a very uncommon cause of scrotal mass in childhood	Urology	2013
Yamashita et al. [1]	Xanthogranulomatous orchitis after blunt testicular trauma mimicking a testicular tumor: a case report and comparison with published cases	Urology Journal	2017
Alazab et al. [22]	Xanthogranulomatous orchitis: rare case with brief literature review	Urology Case Reports	2017
Said et al. [23]	Xanthogranulomatous orchitis: review of the published work, and report of one case	Urology Case Reports	2019
Somani et al. [24]	An unusual case of xanthogranulomatous orchitis: a tumor mimic	Apollo Medicine	2019
e Silva et al. [25]	Xanthogranulomatous orchitis: case report of a rare condition	AME Case Reports	2019
Sharma et al. [26]	Xanthogranulomatous orchitis presenting as a scrotal mass in an elderly male: malignancy or mimicker?	Clinical Medicine Insights: Case Reports	2019
Murshed et al. [27]	A case of xanthogranulomatous orchitis and its preoperative diagnostic challenges	Urology Case Reports	2020
Verma et al. [28]	Scrotal abscess with xanthogranulomatous epididymo-orchitis: a case report of rare diagnostic entity	Tropical Journal of Pathology and Microbiology	2020
Adhlakha et al. [3]	Xanthogranulomatous orchitis mimicking a testicular malignancy: a rare case with brief review of literature	National Journal of Laboratory Medicine	2023
Vijayvergiya et al. [29]	Xanthogranulomatous epididymo-orchitis: a single-institutional case series and systematic review	Indian Journal of Urology	2023

## Conclusions

XGO is a rare condition affecting the testis whose etiology is still uncertain. Though infection plays a considerable role, other causes, such as obstruction to spermatic flow and failure of antegrade flow of sperm, have been seen to initiate an immune response. This immune system activation leads to macrophage infiltration, which plays a significant role in the process of xanthogranulomatous reaction. Through this case report, we aim to emphasize and draw attention to our readers to this rare entity along with its treatment modalities and the differential diagnosis.
